# A Case of Waterhouse-Friderichsen Syndrome Resulting from an Invasive Pneumococcal Infection in a Patient with a Hypoplastic Spleen

**DOI:** 10.1155/2016/4708086

**Published:** 2016-01-28

**Authors:** Kazumasa Emori, Nobuhiro Takeuchi, Junichi Soneda

**Affiliations:** Department of Cardiovascular Surgery, Kobe Tokusyukai Hospital, 1-3-10 Kamitakamaru, Tarumi-ku, Kobe-shi, Hyogo 655-0017, Japan

## Abstract

A 50-year-old male was brought to our emergency department by ambulance with complaints of pain and numbness in both legs. At arrival, purple spots were evident on his neck and face. Examination of the vital sign indicated septic shock. Laboratory data and blood gas analysis revealed disseminated intravascular coagulation, multiple organ failure, and metabolic acidosis. Peripheral blood smears revealed Howell-Jolly bodies, indicating decreased splenic function. A rapid urinary pneumococcal antigen test was also found to be positive. After admission to the intensive care unit, extensive treatment, including polymyxin-B direct hemoperfusion and administration of methylprednisolone and broad spectrum antibiotics was immediately initiated. Despite of our efforts to save his life, the patient died six hours after the arrival. The following day, blood cultures revealed the presence of* Streptococcus pneumoniae*. An autopsy revealed a hypoplastic spleen and a bilateral adrenal hemorrhage, indicating acute adrenal insufficiency caused by sepsis. Finally, the patient was diagnosed with Waterhouse-Friderichsen syndrome. Although severe infection may be seen in the splenectomized patients, it should be noted that patients with a hypoplastic spleen may have acute severe infections. We, therefore, report a case of Waterhouse-Friderichsen syndrome resulting from an invasive pneumococcal infection in a patient with a hypoplastic spleen.

## 1. Background

Waterhouse-Friderichsen syndrome (WFS) is an emergency condition, characterized by fever, cyanosis, bruises, and/or shock [1]. WFS is reported to occur in splenectomized patients, and in patients with congenital asplenia or decreased splenic function [2–5]. Here, we report a rare case of WFS, presenting with purpura fulminans, resulting from an invasive pneumococcal infection.

## 2. Case Presentation

**Figure 1:**
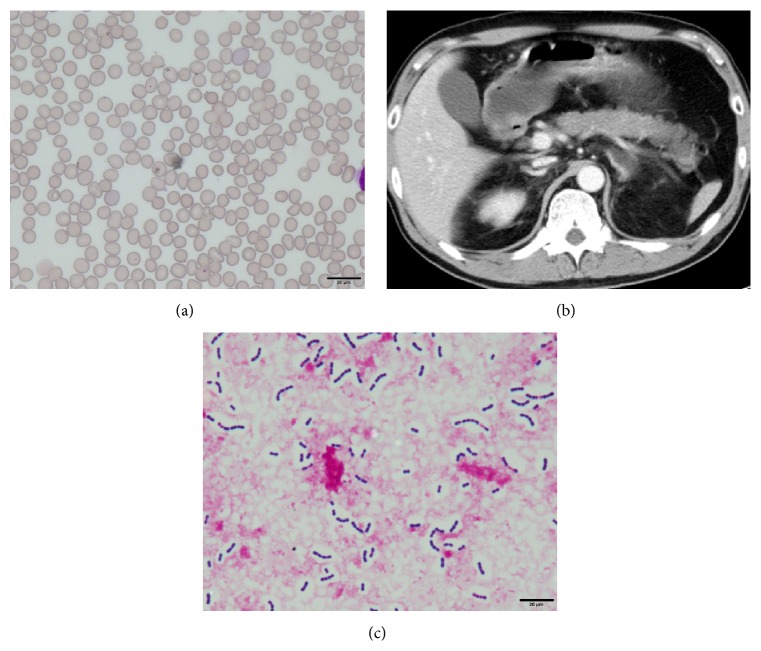
Peripheral blood smear, CT, and blood culture. (a) Peripheral blood smear revealed Howell-Jolly bodies. (b) Contrast CT demonstrated a hypoplastic spleen and retrospective fat tissue stranding around the left adrenal gland. (c) A blood culture revealed streptococci with capsules.

**Figure 2:**
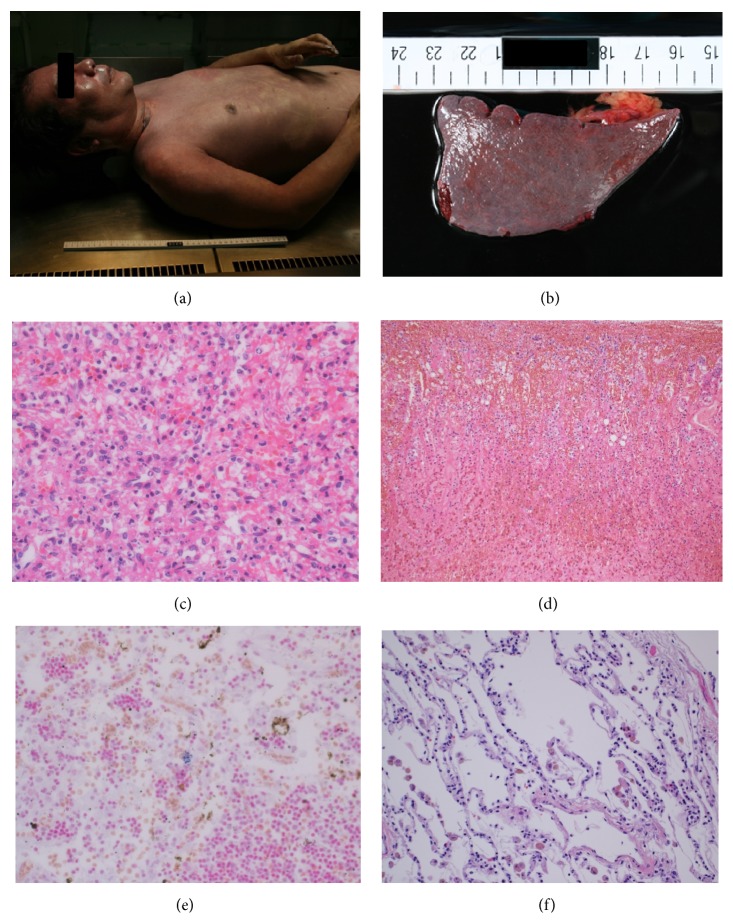
Autopsy analysis. (a) Gross inspection of the entire body revealed purpura fulminans in the face, neck, and chest. (b) Macroscopic analysis of the spleen. The spleen weighted 20 g, with a size of 7.5 × 4.0 × 1.8 cm. (c) Microscopic analysis of the spleen revealed striking neutrophil infiltration. This finding was compatible with acute splenitis, showing evidence of sepsis. (d) Microscopic analysis of the adrenal glands demonstrated hemorrhage in all the layers of the cortex and medulla. Hemosiderin dispositions are apparent. (e, f) Microscopic analysis of the tonsils (e) and lungs (f) revealed streptococci (gram staining).

A 50-year-old male was brought to our hospital by ambulance with complaints of pain and numbness in both legs. According to his wife, he presented with a high-grade fever (39.0°C), pain in both legs, vomiting, and diarrhea on the preceding day. His past medical history also included duodenal ulcer, which was treated by conservative therapy. He was previously diagnosed with fatty liver and an idiopathic leukopenia. According to his wife, the patient had undergone dental treatment three months before. The patient was employed as a system engineer. He had a habit of having 100 cc of whisky and smoking 20 cigarettes daily. Upon arrival, the patient was alert and repeatedly complained of severe pain and numbness in his legs. Furthermore, cyanosis of the whole body was evident, and purple spots were apparent on the patient's neck and face. Examination of the vital signs showed the following results; blood pressure, 85/64 mmHg; heart rate, 113 beats/min; body temperature, 38.2°C; and percutaneous oxygen saturation could not be measured because of apparent peripheral circulatory disturbance. Septic shock was suspected based on the clinical symptoms. An arterial blood gas analysis revealed hypoxemia and metabolic acidosis (pH = 7.280, pO_2_ = 57.0 mmHg, HCO_3_^−^ = 9.2 mmol/L, BE = −15.8 mmol/L). Blood examination revealed leukopenia (3,310  cells/*μ*L), moderately elevated levels of liver enzymes (aspartate aminotransferase 111 IU/L and alanine transaminase 61 IU/L), renal dysfunction (serum creatinine 4.43 mg/dL and blood urea nitrogen 30.3 mg/dL), and coagulation dysfunction (prothrombin time 26%, international normalized ratio 2.42, fibrinogen 37 mg/dL, fibrin and fibrinogen degradation product 86 *μ*g/mL, D-dimer 28 *μ*g/mL, and antithrombin III 58%), and remarkably elevated procalcitonin levels (>100 ng/mL). A peripheral blood analysis revealed Howell-Jolly bodies (Figure 1(a)), indicating decreased splenic function, and a rapid urinary pneumococcal antigen test was also positive. In the emergency department, he was immediately intubated and respiratory management was initiated. At the time of intubation, the pharyngeal mucosa was remarkably reddish and spotted. According to the Japanese Association for Acute Medicine, the disseminated intravascular coagulation (DIC) score was 8; therefore, his condition decisively satisfied the DIC criteria. Contrast computed tomography (CT) did not reveal abscess formation, but a small-sized spleen was noted (Figure 1(b)). Based on the findings of the blood examination along with the clinical symptoms, his condition was compatible with purpura fulminans, and the patient was supposedly in the state of sepsis-associated multiple organ failure as well as acute respiratory distress syndrome (ARDS). Immediately after admitting to the intensive care unit (ICU), a dialysis catheter was inserted to initiate hemodialysis, including continuous hemodiafiltration (CHDF) and polymyxin-B direct hemoperfusion (PMX-DHP). Considerable extracellular fluid along with the transfusion of red blood cells and fresh frozen plasma was administered. Concurrent broad spectrum antibiotics, including imipenem/cilastacin, clindamycin, and piperacillin, were administered. Sivelestat sodium hydrate was initiated for the treatment of ARDS, and 1000 mg of methylprednisolone was administered for the treatment of septic shock. Four hours after the initiation of CHDF and PMX-DHP, his blood pressure suddenly decreased resulting in cardiac arrest. The administration of several doses of adrenaline, as well as chest compressions, restored his heartbeat. However, several minutes after cardiac restoration, cardiac arrest was repeated three to four times. Eventually, he passed away six hours upon arrival. The following day, blood culture results revealed the presence of* Streptococcus* (Figure 1(c)). An autopsy was performed under the consent of his family. Gloss examination of the entire body revealed purple spots across the face, neck, and chest (Figure 2(a)). The autopsy also revealed splenic hypoplasia, with the organ weighting 20 g (Figure 2(b)). Microscopic analysis of the spleen disclosed striking neutrophil infiltration (Figure 2(c)), providing the evidence of sepsis and the analysis of the adrenal glands showed hemorrhage in all layers of the cortex and medulla (Figure 2(d)). Gram staining of the tonsil and lungs provided evidence of streptococcal infection (Figures 2(e) and 2(f)). Thus, the presence of* Streptococcus* in his lungs and tonsils along with bilateral adrenal hemorrhage revealed that the autopsy was consistent with WFS.

## 3. Discussion

It is well- known that splenectomized patients are likely to contract severe infection, which may be fatal; this condition is known as overwhelming post splenectomy infection (OPSI) syndrome [6]. On the other hand, it is less well known that patients with a hypoplastic splenic or decreased splenic function are vulnerable to bacterial infections [7]. The spleen plays a vital role in controlling infections, including bacterial phagocytosis, antigen presentation, antigen production, and production of bacterial opsonin. If spleen function is decreased, it is also known that severe infections can be caused by polysacharide-encapsulated bacteria. In this patient, an enhanced-CT scan demonstrated the small size of the spleen. In general, splenic dysfunction may not only be a result of splenic hypoplasia but also liver cirrhosis, portal hypertension, ulcerative colitis, amyloidosis, sarcoidosis, and collagen diseases. It was revealed at the initial interview that the patient previously had been notified about his decreased peripheral blood cell count resulting from splenic hypoplasia, but no preventative measures had been taken.

Howell-Jolly bodies are seen along with a markedly decreased splenic function. Some reports show that counting Howell-Jolly bodies are a simple and useful measure for assessing splenic function [8] and, in our case, the sample of peripheral blood cells shows the inclusion of Howell-Jolly bodies. Based on this finding along with the evidence provided by the enhanced-CT scan revealing a hypoplastic spleen, the patient was considered to have a decreased splenic function.

Purpura fulminans is a critical syndrome, involving intravascular thrombosis and hemorrhagic infarction of the skin, which rapidly progresses, and is accompanied by vascular collapse or DIC [9]. It is characterized by small vessel thrombosis, leading to tissue necrosis, and is associated with a high mortality rate of approximately 43% [10]. The condition sometimes requires aggressive surgical treatment, including limb amputation, to prevent further dissemination of toxins or to save patients afflicted by purpura fulminans [10]. In our case, with the rapidly worsening condition, surgical treatment was not feasible.

WFS is an emergency condition, characterized by fever, cyanosis, bruises, and/or shock [1]. Even if it is treated, patients with WFS usually die within 24 h after presentation of the syndrome [2]. On autopsy, our case was compatible with the diagnosis of WFS. It is challenging to properly diagnose WFS during the treatment of patients with septic shock and it is supposed that many patients with WFS pass away when left undiagnosed.

Hence, till late, WFS has been considered a post-mortem diagnosis; however, with the development of imaging modalities, including ultrasonography and CT scan, imaging-based diagnosis of adrenal hemorrhage is possible. In general, non-traumatic adrenal hemorrhage revealed by CT scan characteristically presents as being round or oval, with the stranding of periadrenal fat [11]. In our case, the findings of adrenal hemorrhage were not recognized in the settings of the emergency department. In retrospect, it is apparent that the left side adrenal gland was round and accompanied by the stranding of periadrenal fat (Figure 1(b)). The final diagnosis of WFS was made possible by autopsy, with extensive bleeding necrosis of the bilateral adrenal glands. Considering this condition of WFS, acute adrenal insufficiency would be expected and, therefore, the described extensive therapeutic strategy was ineffective in this case. In general, if acute adrenal insufficiency can be diagnosed quickly in ICU settings, early administration of steroidal drugs may, in fact, be life-saving. When treating patients with severe infection diseases, it is important to unravel any existing adrenal insufficiency with the help of imaging modalities, especially CT scan.

## 4. Conclusion

We reported a rare case of Waterhouse-Friderichsen syndrome, presenting with purpura fulminans, resulting from an invasive pneumococcal infection. In the emergency department, it is important to be aware of adrenal insufficiency as well as impaired splenic function whenever patients with sepsis are admitted.
